# Safety and efficacy of teclistamab in systemic immunoglobulin light chain amyloidosis

**DOI:** 10.1038/s41408-023-00950-3

**Published:** 2023-11-27

**Authors:** Rajshekhar Chakraborty, Divaya Bhutani, Mathew S. Maurer, Meera Mohan, Suzanne Lentzsch, Anita D’Souza

**Affiliations:** 1 Columbia University Irving Medical Center, New York, NY USA; 2 Medical College of Wisconsin, Milwaukee, WI USA

**Keywords:** Myeloma, Chemotherapy

**To the editor:**

Bispecific antibodies (bsAbs) targeting B-cell maturation antigen (BCMA) have transformed the landscape of relapsed/refractory (R/R) multiple myeloma, with single-agent response rates of 60–70% in patients who have undergone extensive prior treatments [1–3]. To date, two BCMA-targeting bsAbs have received accelerated approval by the Food and Drug Administration in R/R myeloma-teclistamab and elranatamab. Immunoglobulin light chain (AL) amyloidosis is a related clonal plasma cell disorder, in which, one of the pillars of treatment is effective clone-directed therapy to rapidly achieve a deep hematologic response, ideally a very good partial response (VGPR) or better [4, 5]. Traditionally, clone-directed therapies in AL amyloidosis have been borrowed from successful anti-myeloma therapies, with the most recent example being the anti-CD38 monoclonal antibody daratumumab [6]. Presently, the standard-of-care regimen for newly diagnosed AL amyloidosis is daratumumab in combination with cyclophosphamide-bortezomib-dexamethasone (Dara-CyBorD), which leads to a hematologic complete response (heme-CR) and ≥VGPR rate of about 50 and 80% respectively [6]. However, a critical unmet need remains in the management of patients who have suboptimal hematologic responses to Dara-CyBorD or experience relapse following this regimen. BCMA-targeting bsAbs are an enticing treatment option for AL amyloidosis due to several reasons: (a) they lead to rapid achievement of deep hematologic responses in myeloma, which is critical for achieving organ response in AL amyloidosis; (b) lower incidence of severe cytokine release syndrome (CRS) compared to other T-cell redirecting immunotherapies such as chimeric antigen receptor T-cell therapy. However, there are no prospective or retrospective studies documenting the safety and efficacy of teclistamab in patients with AL amyloidosis, who were excluded from the clinical trials in R/R myeloma. Here, we present data on seven consecutive patients with AL amyloidosis with or without concurrent R/R myeloma from two academic medical centers, who were treated with teclistamab since its FDA approval on 10/25/2022. The data cut-off for follow-up was 10/1/2023. De-identified clinical data can be shared with other investigators upon request to the corresponding author (R.C.).

The demographic and clinical characteristics of our patient are shown in Table 1. Six patients had concurrent AL with R/R myeloma, whereas one had AL alone. The median difference between involved and uninvolved serum-free light chain (dFLC) immediately prior to teclistamab initiation was 20.5 mg/dl (range, 5.3–476.7). Baseline median N-terminal pro brain natriuretic peptide (NT pro-BNP) was 3307 pg/ml (range, 214–19557). Five patients had cardiac involvement and five had renal involvement, with a majority (5/7) having two or more organs involved. The median estimated glomerular filtration rate (eGFR) at baseline was 50 ml/min (range, 5–91), with one patient on peritoneal dialysis. Patients had received a median of 6 prior lines of therapy (range, 2–7), with all patients having prior exposure to anti-CD38 monoclonal antibody (mAb) and 5/7 patients with progressive disease on anti-CD38 mAb. Notably, four patients had exposure to prior BCMA-targeted therapy, including belantamab mafadotin in four and ciltacabtagene autoleucel in one (one patient received both prior to teclistamab). In these four patients, BCMA-targeted therapy was the immediately prior line of treatment preceding teclistamab. At the time of teclistamab initiation, 5/7 patients had disease refractory to the immediately prior line of therapy, with refractoriness defined as less than hematologic partial response (PR) as per standard criteria [7]. At data cut-off, 6/7 patients are alive, with the median follow-up of surviving patients being 3.2 months (range, 1.4–9.0). In the sole patient that died, death happened 40 days after last teclistamab dose due to progressive cardiac deterioration and failure to thrive. With regards to safety, 4/7 patients developed CRS (all grade 1) and none developed immune effector cell-associated neurotoxicity syndrome (ICANS). Tocilizumab was administered in one patient. Notably, all patients were on herpes prophylaxis, 5/7 on Pneumocystis jirovecii pneumonia (PJP) prophylaxis, and 3/7 patients were on primary prophylaxis with intravenous immunoglobulin (IVIG). Two patients developed grade 3 or higher infection-one with grade 3 urinary tract infection (Klebsiella), and one with grade 4 septic shock secondary to gram negative bacilli. There were no infection-related deaths. Only one patient has developed cytopenia till date (grade 3 neutropenia requiring growth factor and grade 3 thrombocytopenia requiring romiplostim plus platelet transfusions).

**Table 1:** Summary of baseline characteristics and response of patients treated with teclistamab.

Patient	Baseline dFLC (mg/dl)	Baseline NT-proBNP (pg/ml) and NYHA Class	Mayo 2004 staging at teclistamab start	Baseline proteinuria (g/24 hours)	Organs Involved	Number of Prior Lines of Therapy	Best Hematologic Response	Organ Response	Vital Status
#1 (60 y/o M)	5.28	3307; NYHA II	II	1.67	Heart Kidney Peripheral Nerves	7	Heme-CR	Cardiac organ response achieved (*Renal response not assessed*)	Alive (in Heme-CR)
#2 (79 y/o F)	5.82	19,557; NYHA III	IIIb	9.76	Heart Kidney	3	Heme-CR	Cardiac and Renal organ response achieved	Alive (in Heme-CR)
#3 (60 y/o F)	20.5	17,035; NYHA I	IIIb^a^	Not evaluable (patient on peritoneal dialysis)	Kidney	5	VGPR	Not evaluable	Alive (in VGPR)
#4 (65 y/o F)	13.55	614; NYHA I	II	0.075	Heart Autonomic Nervous System GI Tract	6	VGPR	Not evaluable	Alive (in VGPR)
#5 (58 y/o F)	476.7	8601; NYHA IV	IIIb	0.075	Heart Kidney GI Tract Musculoskeletal System	7	VGPR	No Response	Dead (in VGPR at the time of death)
#6 (59 y/o F)	21.34	214; NYHA I	I	0.12	Heart	3	VGPR	Not evaluable	Alive (in VGPR)
#7 (71 y/o M)	35.93	1487; NYHA II	IIIa	0.46	Heart Kidney GI Tract	2	VGPR	Cardiac Organ Response Achieved	Alive (in VGPR)

*dFLC* Difference between involved and uninvolved serum free light chain, *NT-proBNP* N-terminal pro-brain natriuretic peptide, *NYHA* New York Heart Association, *CR* complete response, *VGPR* very good partial response.

^a^Although this patient had IIIb disease based on cardiac biomarkers, the elevated cardiac biomarkers were clinically deemed to be secondary to renal failure and patient being on peritoneal dialysis (and not from advanced heart involvement).

**Fig. 1:**
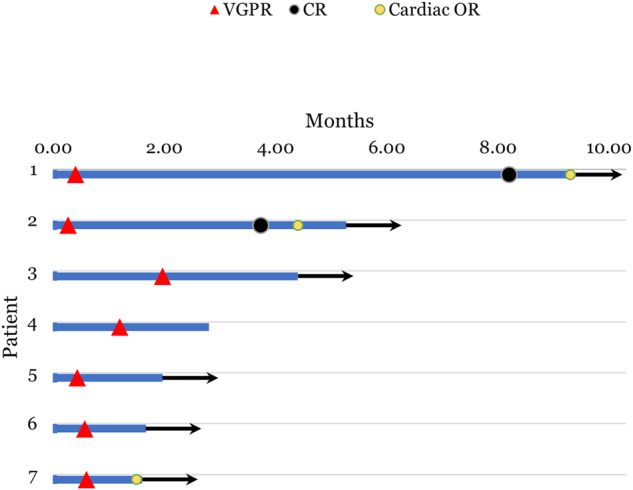
Swimmer’s plot showing the trajectory of individual patients treated with teclistamab. Red triangles represent time at which patients achieved a VGPR (Very Good Partial Response) and black circles represent time of attaining heme-CR (hematologic complete response). Yellow circles represent timing of cardiac organ response. Black arrow at the end of the each bar represents that patient was alive at the time of data cut-off.

Regarding efficacy, all 7 patients (100%) achieved hematologic VGPR or better. Assessment for heme-CR was limited by absence of data on urine electrophoresis and immunofixation in 4/7 patients. However, among three patients with available data on serum and urine electrophoresis/immunofixation, two had achieved heme-CR. Responses were rapid, as evidenced by median time to VGPR from teclistamab initiation of 0.6 months (range, 0.3–1.9). At the 1-month landmark from treatment initiation, 6/7 patients had achieved a stringent dFLC response (defined by dFLC<1 mg/dl [8]). Among four patients that were evaluable for cardiac organ response (i.e. cardiac involvement with baseline NT-proBNP > 650 pg/ml), three achieved a cardiac response. Among two patients evaluated for renal organ response (i.e. 24-hour urine protein ≥0.5 g), one has achieved a renal response and the response has not been assessed yet in one patient. None of the patients had hematologic relapse or progression at the latest follow-up. A swimmer’s plot with the treatment course of each patient is shown in Fig. 1.

In conclusion, our series shows that teclistamab can be used safely in the treatment of selected patients with systemic AL amyloidosis. We observed rapid and deep hematologic responses with minimal acute toxicity concerning CRS and ICANS. Given the presence of baseline cardiac dysfunction and hypotension is many patients with cardiac AL amyloidosis, CRS is a potential concern with the use of these drugs. As we show here, only grade 1 CRS was seen in 4/7 patients and it did not lead to any adverse outcomes. Notably, previous literature on teclistamab in AL amyloidosis remains limited, with a single case report lacking comprehensive data on hematologic or organ responses [9]. Currently, treatment options are limited for transplant-ineligible patients with AL amyloidosis who are failed by frontline Dara-CyBorD. The commonly used second-line treatments that are supported by prospective data are pomalidomide, ixazomib, and bendamustine. The proportion of patients who achieve VGPR or better with these agents is modest, and ranges from 18–38% for pomalidomide [10, 11], 36–43% with ixazomib [12, 13], and 23% with bendamustine [14]. Furthermore, these data pre-date the use of Dara-CyBorD frontline therapy, and hence, it remains unclear whether these response rates would hold up in patients after exposure to Dara-CyBorD. The only RCT that exists in the relapsed/refractory setting comparing ixazomib-dexamethasone to physician’s choice did not meet its primary endpoint, with a ≥VGPR rate of 36 and 32% in investigational and control arms respectively [13]. Among patients harboring a t(11;14) translocation, the BCL2 inhibitor venetoclax has promising retrospective data, with ≥VGPR rate of more than 70% [15], and prospective studies on venetoclax are currently accruing. In contrast, our case series with teclistamab revealed an impressive ≥VGPR rate of 100%, warranting further prospective investigation in AL amyloidosis. Since patients with AL amyloidosis have a lower disease burden in bone marrow, it is plausible that teclistamab will have a higher response rate in AL amyloidosis compared to multiple myeloma, since a trend toward greater response rate was noted in patients with BMPC < 30% in the MajesTEC-1 trial [1]. However, questions regarding the durability of hematologic response and long-term outcomes with teclistamab remain unanswered, given the absence of extended follow-up data for BCMA bsAbs. Additionally, it remains unclear as to whether patients with systemic AL amyloidosis can tolerate high-grade CRS or ICANS, which can potentially happen in patients with high baseline bone marrow plasma cell burden. Finally, considering the cumulative risk of infection-related complications with prolonged treatment [16, 17], future prospective studies of teclistamab in AL amyloidosis should explore fixed-duration therapy accompanied by robust infection prophylaxis measures.
